# The human central nervous system discharges carbon dioxide and lactic acid into the cerebrospinal fluid

**DOI:** 10.1186/s12987-019-0128-7

**Published:** 2019-03-29

**Authors:** Tetsuya Akaishi, Eiko Onishi, Michiaki Abe, Hiroaki Toyama, Kota Ishizawa, Michio Kumagai, Ryosuke Kubo, Ichiro Nakashima, Masashi Aoki, Masanori Yamauchi, Tadashi Ishii

**Affiliations:** 1 Department of Education and Support for Regional Medicine, Tohoku University Hospital, Seiryo-machi 1-1, Aoba-ku, Sendai, Miyagi 980-8574 Japan; 2 Department of Neurology, Tohoku University Graduate School of Medicine, Sendai, Japan; 3 Department of Anesthesiology and Perioperative Medicine, Tohoku University Hospital, Sendai, Japan; 4 Tohoku Medical Megabank Organization, Tohoku University, Sendai, Japan; 5 Department of Neurology, Tohoku Medical and Pharmaceutical University, Sendai, Japan

**Keywords:** Brain metabolism, Carbon dioxide, Cerebrospinal fluid, Functional role, Lactic acid

## Abstract

**Background:**

The central nervous system was previously thought to draw oxygen and nutrition from the arteries and discharge carbon dioxide and other metabolic wastes into the venous system. At present, the functional role of cerebrospinal fluid in brain metabolism is not fully known.

**Methods:**

In this prospective observational study, we performed gas analysis on venous blood and cerebrospinal fluid simultaneously acquired from 16 consecutive preoperative patients without any known neurological disorders.

**Results:**

The carbon dioxide partial pressure (pCO_2_) (p < 0.0001) and lactic acid level (p < 0.001) in the cerebrospinal fluid were significantly higher than those in the peripheral venous blood, suggesting that a considerable proportion of metabolic carbon dioxide and lactic acid is discharged from the central nervous system into the cerebrospinal fluid. The oxygen partial pressure (pO_2_) was much higher in the cerebrospinal fluid than in the venous blood, corroborating the conventional theory of cerebrospinal fluid circulatory dynamics. The pCO_2_ of the cerebrospinal fluid showed a strong negative correlation with age (R = − 0.65, p = 0.0065), but the other studied variables did not show significant correlation with age.

**Conclusion:**

Carbon dioxide and lactic acid are discharged into the circulating cerebrospinal fluid, as well as into the venules. The level of carbon dioxide in the cerebrospinal fluid significantly decreased with age.

## Background

The central nervous system (CNS) is commonly believed to draw oxygen and nutrition from the arterial system and discharge carbon dioxide (CO_2_) and metabolic wastes, such as lactic acid, into the venous system. However, in addition to this conventional metabolic pathway directly from artery to vein, cerebrospinal fluid (CSF) is derived from the arterial system and drained into the venous system, with a turnover rate of three to five times per day [1, 2]. The CSF is known to function as a shock absorber for the CNS, protecting it from external impact [3–5], but its physiological functions and its role in CNS metabolism are not fully known.

Recently, researchers discovered a CNS drainage system passing through the CSF-filled para-vascular space and eventually leading to the dural lymphatic vascular system and cervical lymph nodes [6, 7]. This para-vascular CSF flux is thought to allow the exchange of water and solutes between the interstitial fluid (ISF) of the parenchyma and the CSF [8–10]. One subsequent experiment showed that this para-vascular CSF–ISF exchange is facilitated by arterial pulsation [11], while another revealed that it allows metabolic wastes in the brain and spine to be drained into the circulating CSF along with the pulsatile bulk flow [12]. As a result, the circulating CSF probably contains unknown levels of metabolites discharged from the ISF of the brain parenchyma via the para-vascular space.

However, to our knowledge, no human studies have compared dissolved substances, including metabolites such as CO_2_ and lactic acid, between simultaneously-obtained samples of CSF and venous blood. In the present study, we compared dissolved solutes, namely oxygen, CO_2_, electrolytes, and lactic acid, between simultaneously-acquired CSF and venous blood samples from preoperative subjects with no known neurological diseases. In so doing, we aimed to ascertain the solute gradient between the CSF and venous blood, and to achieve new insights into the role of CSF in the CNS drainage system.

## Materials and methods

### Ethics statement

This study was performed in compliance with the Code of Ethics of the World Medical Association (Declaration of Helsinki; 1989). Blood and CSF samples were collected at the Tohoku University Hospital (Sendai, Japan). The study was approved by the Institutional Review Board of Tohoku University Hospital (IRB approval number: 2018-1-475). Written informed consent was obtained from all enrolled subjects.

### Subject enrollment criteria

All enrolled subjects were preoperative patients awaiting lumbar anesthesia, and all were aged ≥ 18 years. The following exclusion criteria were applied: (1) pregnancy, (2) severe spinal canal stenosis, (3) diagnosed neurological disorders, including dementia, (4) brain or spinal cord lesions.

Based on these criteria, we selected 16 consecutive patients in December 2018. They were awaiting the following types of procedure: knee joint surgery, skin biopsy, hemorrhoid surgery, etc. All procedures were unrelated to the brain and spinal cord.

### Blood and CSF sampling

Venous blood and CSF samples were extracted from each patient in the operating room of Tohoku University Hospital during the lumbar anesthesia procedure. Venous blood was extracted from each arm, while CSF was extracted at the lumbar level. Patients were conscious and received no supplemental oxygen during sample collection. None of the patients had any history of respiratory disease with ventilatory impairment. Both venous blood and CSF were extracted using a 5-cm^3^ blood collection syringe designed for use in blood gas analysis. From each patient, 1 cm^3^ of venous blood and CSF were extracted. Because the samples were aspirated directly from the needle, neither was exposed to room air. Thus, it is unlikely that the room air or the time period between collection and sample measurement affected the data. The extracted venous blood and CSF were subjected to gas analysis within 10 min of sample collection.

### Gas analysis device and measured variables

All analysis was performed using the same device (ABL800 FLEX blood gas analyzer; Radiometer, Brønshøj, Denmark) in the operating room of Tohoku University Hospital. The following variables were measured: pH, oxygen partial pressure (pO_2_), CO_2_ partial pressure (pCO_2_), standard base excess (SBE), bicarbonate (HCO_3_^−^), sodium ion (Na^+^), potassium ion (K^+^), chloride ion (Cl^−^), anion gap (AG), glucose level, lactic acid level, and total bilirubin level.

### Statistical analysis

Gas analysis data were compared between the venous blood and CSF of each subject using either the paired *t* test or the Wilcoxon signed-rank test, depending on whether the data were normally distributed. The lactic acid level of the venous blood was abnormal (4.3 mmol/L) in one subject; a test of outliers confirmed that this result was an outlier (p < 0.0001). Thus, the lactic acid data from this subject were not included in the present study because the measured level suggested that the patient had lactic acidosis that could have biased the data interpretation.

Because multiple variables were compared simultaneously, p-value < 0.01 was regarded as statistically significant. Statistical analyses were conducted using either SPSS Statistics Base 22 software (IBM, Armonk, NY, USA) or MATLAB R2015a (MathWorks, Natick, MA, USA).

## Results

### Demographics and clinical information

The 16 enrolled subjects (10 men, six women) had a mean age of 59.5 years (range 25–87) and a mean body mass index (BMI) of 23.9 (range 16.5–38.9). Eight underwent lumbar anesthesia for hemorrhoid surgery, six for skin biopsy, and two for joint surgery.

### Comparisons of gas analysis between venous blood and CSF

The gas analysis results of the venous blood and CSF samples are listed and compared in Table 1. The mean pO_2_ (p < 0.0001, paired t-test), pCO_2_ (p < 0.0001, paired t-test), and lactic acid level (p < 0.001, Wilcoxon signed-rank test) were significantly higher in the CSF samples than in the venous blood samples.

**Table 1:** Solute and pH levels in the venous blood and CSF samples simultaneously extracted from 16 subjects

	Venous blood	CSF	p-value
pH	7.40 ± 0.03	7.33 ± 0.04	< 0.0001
pO_2_ [mmHg]	53.7 ± 12.1	77.0 ± 8.7	< 0.0001
pCO_2_ [mmHg]	41.3 ± 5.1	48.5 ± 3.9	< 0.0001
SBE [mmol/L]	1.03 ± 2.31	− 0.21 ± 1.81	0.0266
HCO_3_^−^ [mmol/L]	25.3 ± 2.4	24.9 ± 1.5	0.429
Na^+^ [mmol/L]	139.7 ± 2.0	140.1 ± 1.6	0.343
K^+^ [mmol/L]	3.78 ± 0.38	2.73 ± 0.10	< 0.0001
Cl^−^ [mmol/L]	103.7 ± 3.1	118.1 ± 2.7	< 0.0001
AG (K^+^) [mmol/L]	14.0 ± 1.5	− 0.1 ± 2.7	< 0.0001
Lac [mmol/L]	1.01 ± 0.35	1.46 ± 0.20	0.0004
t-Bil [mg/dL]	0.92 ± 0.62	0.02 ± 0.04	< 0.0001

The mean pO_2_ was significantly higher in the CSF than in venous blood samples, as were the mean pCO_2_ and lactic acid levels, suggesting that these metabolic wastes were discharged from the central nervous system into the CSF via some drainage system. All p-values are the result of a paired t-test as a parametric test or a Wilcoxon signed-rank test as a non-parametric test

AG, anion gap; Cl^−^, chloride ion; CSF, cerebrospinal fluid; HCO_3_^−^, bicarbonate; K^+^, potassium ion; Na^+^, sodium ion; pCO_2_, carbon dioxide partial pressure; Lac, lactic acid; pO_2_, oxygen partial pressure; SBE, standard base excess; t-Bil, total bilirubin

**Fig. 1:**
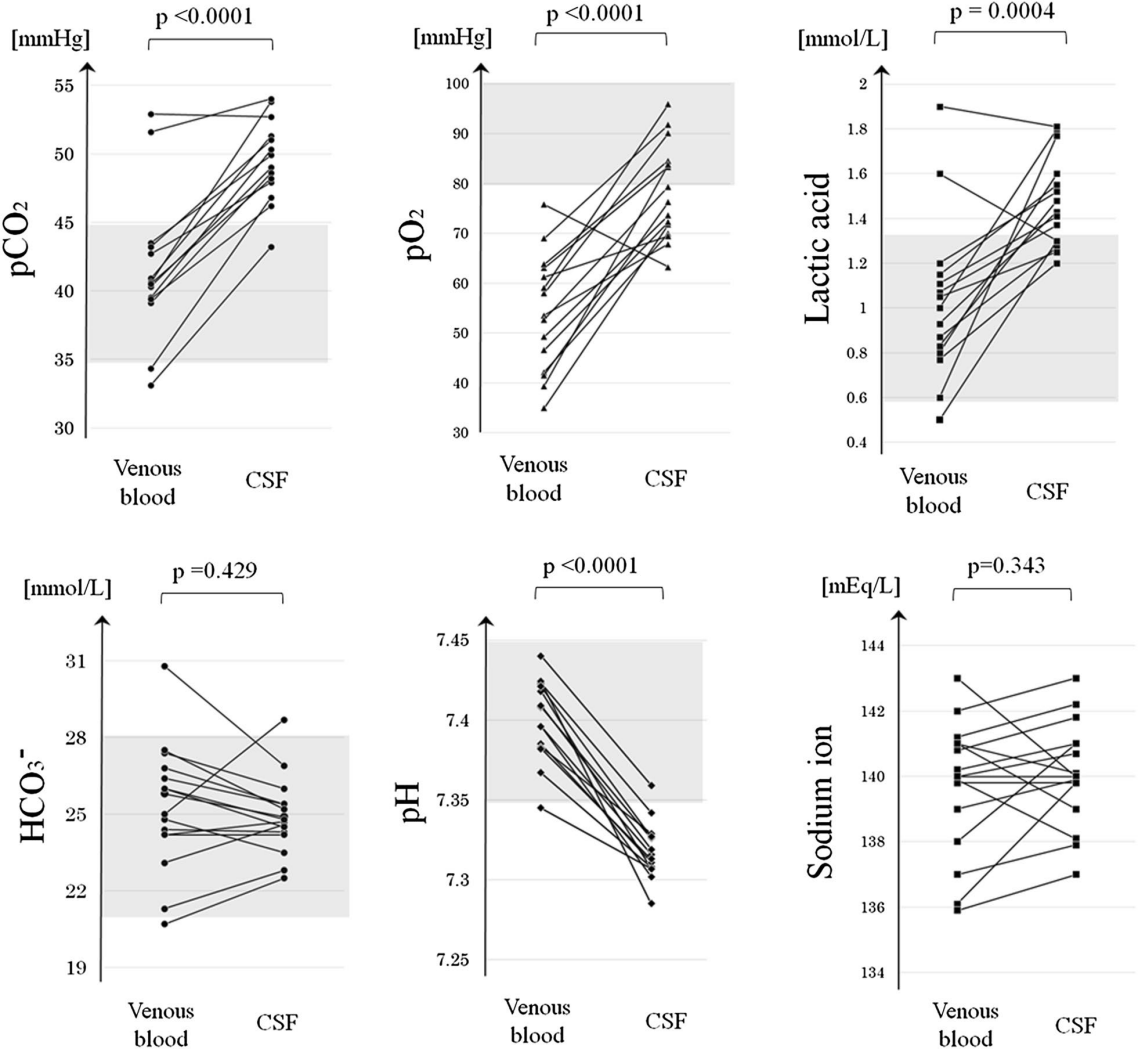
Comparisons of solute levels and pH between venous blood and CSF. The distribution of pO_2_ was higher in CSF than in venous blood, as were those of pCO_2_ and lactic acid, indicating that the brain and spine discharge CO_2_ and lactic acid into the CSF. The CSF was significantly more acidic than the venous blood. Levels of HCO^3−^ and Na^+^ ions did not differ significantly between the venous blood and CSF. The grey-filled areas show the generally accepted normal ranges in arterial blood. All p-values are the result of a paired t-test. CSF, cerebrospinal fluid; HCO_3_^−^, bicarbonate ion; pCO_2_, carbon dioxide partial pressure; pO_2_, oxygen partial pressure

To visually confirm that the dissolved levels of these solutes were actually higher in CSF than in venous blood in most enrolled subjects, the paired distributions of the pO_2_, pCO_2_, and lactic acid level were depicted (Fig. 1). The distributions of all three solutes were higher in the CSF than in the venous blood of most enrolled patients.

The mean pH, K^+^ level, and AG were significantly lower in CSF than in venous blood (p < 0.0001 in all cases, paired t-test). Meanwhile, the mean Na^+^ level and HCO^3−^ level did not differ between CSF and venous blood (p ≥ 0.10 in both cases, paired t-test). The mean Cl^−^ level was significantly higher in CSF than in venous blood (p < 0.0001, paired t-test).

### Age-dependency of solute levels in CSF

**Fig. 2:**
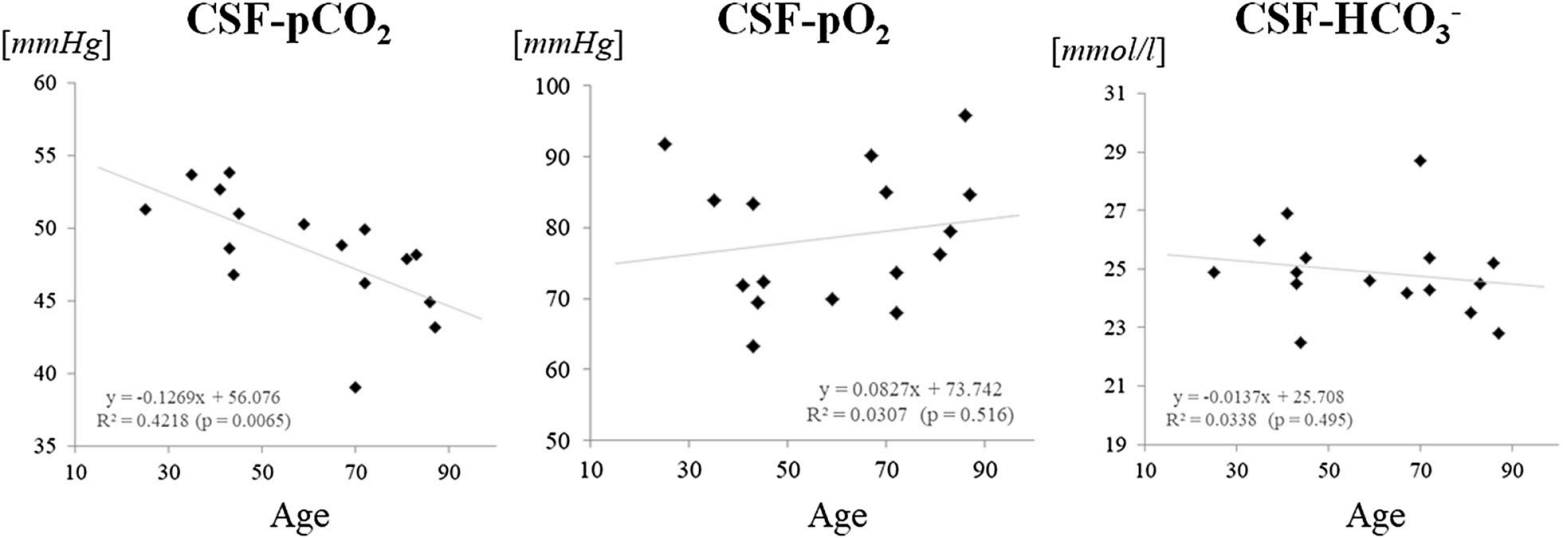
Scatter plots of the CSF solute levels by age. The pCO_2_ in the CSF showed a significant negative correlation with age (R = − 0.65, p = 0.0065), while no other measured variables showed any significant correlations with age. CSF, cerebrospinal fluid; HCO_3_^−^, bicarbonate; pCO_2_, carbon dioxide partial pressure; pO_2_, oxygen partial pressure

The pCO_2_, pO_2_, and HCO_3_^−^ levels in CSF were evaluated for correlation with age. Figure 2 shows that pCO_2_ in CSF showed a significant negative correlation with age (Pearson’s correlation coefficient [R] = − 0.650, p = 0.0065), while pO_2_ and HCO_3_^−^ in CSF showed no significant correlation with age (R < 0.20, p ≥ 0.10 in both cases). None of the following factors showed any significant correlation with age: pCO_2_ in venous blood, lactic acid in venous blood, and lactic acid in CSF (p ≥ 0.10 in all cases).

## Discussion

In the present report, the levels of pCO_2_ and lactic acid were higher in CSF than in venous blood, suggesting that the CNS discharges CO_2_ and lactic acid into the CSF. Furthermore, the levels of pCO_2_ in the CSF significantly decreased by age, even though those in the venous blood did not change, suggesting that the efficiency of CO_2_ discharge from the brain parenchyma into the CSF decreases with age. However, this was an observational study, so the exact mechanism of CO_2_ and lactate discharge into CSF remains unclear. Nonetheless, our findings may offer new insight into the role of CSF in the drainage system of the CNS.

### Movement of lactic acid in the CNS

The movement of lactic acid across the cell membrane is mainly mediated by proton-linked mono-carboxylate transporters (MCTs) [13–15], which are widely distributed throughout the body of all living organisms, even on circulating blood cells and brain astrocytes [16, 17]. In total, 14 types of MCTs (MCT 1–14) have been identified, with each type showing specific tissue distribution across the body [18, 19]. Within the CNS, MCT 1, MCT 2, and MCT 4 are known to be expressed by ependymal cells and astrocytes [20, 21], and several types of MCTs are also suggested to be expressed by the endothelial cells of micro-vessels and choroid plexus epithelial cells [22, 23]. The ependymal cell layer does not contain tight junctions and solutes are thought to freely diffuse through the intercellular gaps, suggesting that MCTs are not necessarily required for the ISF-CSF exchange. Meanwhile, because the microvascular endothelial cells usually form tight junctions, exchange of lactate between blood and CSF requires MCTs. Because most MCTs function as gradient-dependent transporters regulated by the gradient of lactate and protons across the membrane [24, 25], the observed gradient of lactate level between the venous blood and the CSF in this study would be rationally explained by the discharge from ISF into CSF, rather than the transport via the choroid plexus. Several studies have suggested that MCTs are predominantly expressed by ependymal cells on the basolateral side, while others have observed MCTs on the apical side, facing the CSF [26, 27]. Thus, in addition to the drainage system via para-vascular CSF flux, ependymal cells may also play a role in the discharge of lactic acid from the brain parenchymal ISF into the CSF.

### Diffusion of CO_2_ in the CNS

The CO_2_ in the brain parenchyma probably discharges into the circulating CSF via gradient-based diffusion. Previously, it was assumed that dissolved gases were allowed to diffuse across cell membranes almost without restriction [28]. Later studies showed that the movement of CO_2_ across the cell membrane is somewhat restricted, because the unstirred layer in the membrane vicinity functions as diffusional barrier [29, 30]. More recently, the degree of cholesterol content in the phospholipid membrane was shown to be the primary regulator of membrane permeability to CO_2_ [31]. Although the permeability of CO_2_ would be surely restricted to some extent by the glial cells that delineate the ISF from the CSF, ependymal cells and perivascular astrocytic endfeet form a looser barrier than microvascular endothelial cells that form tight junctions. Because there are gaps of varying tightness in the ependymal cell lining and astrocytic endfeet, CO_2_ would be able to easily diffuse from the brain parenchymal ISF into the CSF [5, 32]. Consequently, the ISF of the brain parenchyma, in which the metabolites from neurons are dissolved, can be regarded as being directly connected with the CSF as a continuum. Considered together with the findings of the present study, it follows that CO_2_ from the CNS drains into the CSF, possibly via para-vascular CSF flux or ependymal gap junctions, as well as into the venules via capillaries. Therefore, because the CSF turns over three to five times per day [1, 2], it is likely that the circulating CSF makes a significant contribution to the removal of CO_2_ and lactic acid from the CNS.

### Age-dependent decreases in CSF pCO_2_

Another notable finding of this study was that the pCO_2_ in the CSF significantly decreased with age, whereas that in the venous blood did not, suggesting that CO_2_ discharge from the CNS into the CSF gradually decreases with age, or that the turnover rate of circulating CSF increases with age. However, the CSF turnover rate is known to significantly decrease with age [33], so the age-dependent decrease in CSF pCO_2_ cannot be explained in these terms. Therefore, it is likely that either CO_2_ production within the brain parenchyma or the efficiency of CO_2_ discharge into the CSF decreases with age. Future research is warranted to evaluate the efficiency of CO_2_ discharge into the CSF in patients with neurological diseases of unknown cause.

### Possible methodological errors

Finally, we shall consider whether the absence of blood cells in the CSF may have affected the measured data and derived conclusions. The gas analyzer used (ABL800 FLEX) measures pH and pCO_2_ using potentiometry, which is based on the Nernst equation; pO_2_ and lactic acid were measured using amperometry. One previous study validated and recommended the use of gas analysis to measure pH in pleural effusion samples [34], so gas analyzers can reliably measure pH and dissolved partial pressures, regardless of whether the sample contains blood cells. In further support of this conclusion, Na^+^ concentrations measured using potentiometry did not differ significantly between venous blood and CSF, nor did HCO_3_^−^ levels calculated on the basis of pCO_2_ and pH using the Henderson–Hasselbalch equation (p ≥ 0.10 in both cases, paired t-test).

## Limitations

The principle limitation of the present study was the small sample size. In addition, the study only reported an observation of physiological phenomena, the exact molecular mechanism of which was not elucidated. The clinical significance of the age-dependent decrease in CSF pCO_2_ also remains unclear. To further characterize the association between CO_2_ discharge into the CSF and various neurological disorders, future researchers should compare controls with patients who have such disorders. Another limitation is that gas analysis of arterial blood was not performed in the present study, so it is not clear whether the enrolled subjects had normal blood gas levels in their arterial blood. Finally, peripheral venous blood was extracted from the arms in the present study, rather than from the venous sinuses around the arachnoid granulations. We did not confirm experimentally that the levels of solutes and dissolved gases in venous blood from the arms were comparable to those in the venous sinuses. To determine the concentration gradient between CSF and venous blood, samples must be acquired from adjacent sites.

## Conclusion

The present results demonstrated that CO_2_ and lactic acid are discharged from the human CNS into the circulating CSF. The pCO_2_ of the CNS may decrease with age, even though that in venous blood does not. The clinical significance of such a CO_2_-drainage system in the CNS has not yet been elucidated and should be addressed in future research.

## Abbreviations

AG: anion gap
Cl^−^: chloride ion
CNS: central nervous system
CO_2_: carbon dioxide
CSF: cerebrospinal fluid
HCO^3−^: bicarbonate
K^+^: potassium ion
Na^+^: sodium ion
pCO_2_: carbon dioxide partial pressure
pO_2_: oxygen partial pressure

## References

[1] Tumani H, Huss A, Bachhuber F (2017). The cerebrospinal fluid and barriers—anatomic and physiologic considerations. Handb Clin Neurol..

[2] Simon MJ, Iliff JJ (2016). Regulation of cerebrospinal fluid (CSF) flow in neurodegenerative, neurovascular and neuroinflammatory disease. Biochem Biophys Acta.

[3] Han CY, Backous DD (2005). Basic principles of cerebrospinal fluid metabolism and intracranial pressure homeostasis. Otolaryngol Clin North Am.

[4] Spector R, Robert Snodgrass S, Johanson CE (2015). A balanced view of the cerebrospinal fluid composition and functions: focus on adult humans. Exp Neurol.

[5] Jimenez AJ, Dominguez-Pinos MD, Guerra MM, Fernandez-Llebrez P, Perez-Figares JM (2014). Structure and function of the ependymal barrier and diseases associated with ependyma disruption. Tissue Barriers..

[6] Louveau A, Smirnov I, Keyes TJ, Eccles JD, Rouhani SJ, Peske JD (2015). Structural and functional features of central nervous system lymphatic vessels. Nature.

[7] Aspelund A, Antila S, Proulx ST, Karlsen TV, Karaman S, Detmar M (2015). A dural lymphatic vascular system that drains brain interstitial fluid and macromolecules. J Exp Med.

[8] Hladky SB, Barrand MA (2014). Mechanisms of fluid movement into, through and out of the brain: evaluation of the evidence. Fluids Barriers CNS..

[9] Hladky SB, Barrand MA (2016). Fluid and ion transfer across the blood–brain and blood–cerebrospinal fluid barriers; a comparative account of mechanisms and roles. Fluids Barriers CNS..

[10] Nedergaard M (2013). Neuroscience. Garbage truck of the brain. Science.

[11] Iliff JJ, Wang M, Zeppenfeld DM, Venkataraman A, Plog BA, Liao Y (2013). Cerebral arterial pulsation drives paravascular CSF-interstitial fluid exchange in the murine brain. J Neurosci.

[12] Iliff JJ, Wang M, Liao Y, Plogg BA, Peng W, Gundersen GA, et al. A paravascular pathway facilitates CSF flow through the brain parenchyma and the clearance of interstitial solutes, including amyloid beta. Science translational medicine. 2012;4(147):147ra11. 10.1126/scitranslmed.3003748 PMC3551275 22896675

[13] Iliff JJ, Wang M, Liao Y, Plogg BA, Peng W, Gundersen GA (2012). A paravascular pathway facilitates CSF flow through the brain parenchyma and the clearance of interstitial solutes, including amyloid beta. Sci Transl Med..

[14] Bonen A (2000). Lactate transporters (MCT proteins) in heart and skeletal muscles. Med Sci Sports Exerc.

[15] Juel C, Halestrap AP (1999). Lactate transport in skeletal muscle—role and regulation of the monocarboxylate transporter. J Physiol.

[16] Broer S, Rahman B, Pellegri G, Pellerin L, Martin JL, Verleysdonk S (1997). Comparison of lactate transport in astroglial cells and monocarboxylate transporter 1 (MCT 1) expressing *Xenopus laevis* oocytes. Expression of two different monocarboxylate transporters in astroglial cells and neurons. J Biol Chem..

[17] Meredith D, Bell P, McClure B, Wilkins R (2002). Functional and molecular characterisation of lactic acid transport in bovine articular chondrocytes. Cell Physiol Biochem.

[18] Garcia CK, Brown MS, Pathak RK, Goldstein JL (1995). cDNA cloning of MCT2, a second monocarboxylate transporter expressed in different cells than MCT1. J Biol Chem.

[19] Bonen A, Heynen M, Hatta H (2006). Distribution of monocarboxylate transporters MCT1–MCT8 in rat tissues and human skeletal muscle. Appl Physiol Nutr Metab..

[20] Pierre K, Pellerin L (2005). Monocarboxylate transporters in the central nervous system: distribution, regulation and function. J Neurochem.

[21] Tomioka NH, Nakamura M, Doshi M, Deguchi Y, Ichida K, Morisaki T (2013). Ependymal cells of the mouse brain express urate transporter 1 (URAT1). Fluids Barriers CNS..

[22] Koehler-Stec EM, Simpson IA, Vannucci SJ, Landschulz KT, Landschulz WH (1998). Monocarboxylate transporter expression in mouse brain. Am J Physiol.

[23] Philp NJ, Yoon H, Lombardi L (2001). Mouse MCT3 gene is expressed preferentially in retinal pigment and choroid plexus epithelia. Am J Physiol Cell Physiol.

[24] Payen VL, Hsu MY, Radecke KS, Wyart E, Vazeille T, Bouzin C (2017). Monocarboxylate transporter MCT1 promotes tumor metastasis independently of its activity as a lactate transporter. Can Res.

[25] Le Floch R, Chiche J, Marchiq I, Naiken T, Ilc K, Murray CM (2011). CD147 subunit of lactate/H+ symporters MCT1 and hypoxia-inducible MCT4 is critical for energetics and growth of glycolytic tumors. Proc Natl Acad Sci USA.

[26] Gerhart DZ, Enerson BE, Zhdankina OY, Leino RL, Drewes LR (1997). Expression of monocarboxylate transporter MCT1 by brain endothelium and glia in adult and suckling rats. Am J Physiol.

[27] Pellerin L, Bergersen LH, Halestrap AP, Pierre K (2005). Cellular and subcellular distribution of monocarboxylate transporters in cultured brain cells and in the adult brain. J Neurosci Res.

[28] Endeward V, Al-Samir S, Itel F, Gros G (2014). How does carbon dioxide permeate cell membranes? A discussion of concepts, results and methods. Front Physiol.

[29] Missner A, Kugler P, Saparov SM, Sommer K, Mathai JC, Zeidel ML (2008). Carbon dioxide transport through membranes. J Biol Chem.

[30] Gutknecht J, Bisson MA, Tosteson FC (1977). Diffusion of carbon dioxide through lipid bilayer membranes: effects of carbonic anhydrase, bicarbonate, and unstirred layers. J Gen Physiol.

[31] Itel F, Al-Samir S, Oberg F, Chami M, Kumar M, Supuran CT (2012). CO2 permeability of cell membranes is regulated by membrane cholesterol and protein gas channels. FASEB J.

[32] Roales-Bujan R, Paez P, Guerra M, Rodriguez S, Vio K, Ho-Plagaro A (2012). Astrocytes acquire morphological and functional characteristics of ependymal cells following disruption of ependyma in hydrocephalus. Acta Neuropathol.

[33] Chen CP, Chen RL, Preston JE (2010). The influence of cerebrospinal fluid turnover on age-related changes in cerebrospinal fluid protein concentrations. Neurosci Lett.

[34] Colice GL, Curtis A, Deslauriers J, Heffner J, Light R, Littenberg B (2000). Medical and surgical treatment of parapneumonic effusions: an evidence-based guideline. Chest.

